# PD-L1 targeted peptide demonstrates potent antitumor and immunomodulatory activity in cancer immunotherapy

**DOI:** 10.3389/fimmu.2024.1367040

**Published:** 2024-04-30

**Authors:** Yulai Liang, Huazao Luo, Xue Li, Shuang Liu, Arslan Habib, Baoxiu Liu, Jiansheng Huang, Jingbo Wang, Han Yi, Bo Hu, Liuhai Zheng, Jun Xie, Naishuo Zhu

**Affiliations:** ^1^ Laboratory of Molecular Immunology, State Key Lab of Genetic Engineering, School of Life Sciences, Fudan University, Shanghai, China; ^2^ Renal Division, Department of Internal Medicine, Xinhua Hospital, Shanghai Jiao Tong University School of Medicine, Shanghai, China; ^3^ Institute of Biomedical Sciences, School of Life Sciences, Fudan University, Shanghai, China

**Keywords:** immunotherapy, immune checkpoint inhibitors, PPL-C, PD-L1 binding peptide, colon cancer

## Abstract

**Background:**

In recent years, immunotherapy has been emerging as a promising alternative therapeutic method for cancer patients, offering potential benefits. The expression of PD-L1 by tumors can inhibit the T-cell response to the tumor and allow the tumor to evade immune surveillance. To address this issue, cancer immunotherapy has shown promise in disrupting the interaction between PD-L1 and its ligand PD-1.

**Methods:**

We used mirror-image phage display technology in our experiment to screen and determine PD-L1 specific affinity peptides (PPL-C). Using CT26 cells, we established a transplanted mouse tumor model to evaluate the inhibitory effects of PPL-C on tumor growth in vivo. We also demonstrated that PPL-C inhibited the differentiation of T regulatory cells (Tregs) and regulated the production of cytokines.

**Results:**

In vitro, PPL-C has a strong affinity for PD-L1, with a binding rate of 0.75 μM. An activation assay using T cells and mixed lymphocytes demonstrated that PPL-C inhibits the interaction between PD-1 and PD-L1. PPL-C or an anti-PD-L1 antibody significantly reduced the rate of tumor mass development in mice compared to those given a control peptide (78% versus 77%, respectively). The results of this study demonstrate that PPL-C prevents or retards tumor growth. Further, immunotherapy with PPL-C enhances lymphocyte cytotoxicity and promotes proliferation in CT26-bearing mice.

**Conclusion:**

PPL-C exhibited antitumor and immunoregulatory properties in the colon cancer. Therefore, PPL-C peptides of low molecular weight could serve as effective cancer immunotherapy.

## Introduction

Immune monitoring of newly developed cancer cells is one of the body's defenses against malignancies (1, 2). Through the overexpression of detrimental immunologic regulators, these cells avoid immunologic surveillance. The immune system is often blocked from attacking self-antigens by these regulators, which act as checkpoints. Immune escape is the term for what happens when cancer cells overproduce these regulators and use strategies to dodge the immune system, allowing them to grow unchecked (1, 3). To stimulate and increase tumor-responsive T cells, many immunotherapeutic strategies have been developed. However, cancers can avoid immunological eradication even with potent antitumor T cell feedback. One way is the production of a protein known as programmed death ligand 1 (PD-L1, B7-H1) by tumor cells, which suppressess immune responses by preventing effector T cell growth or survival (4). PD-L1 is greatly expressed in tumor cells or in the tumor microenvironment (TME), which may indicate that lymphocytes have entered the tumor (5, 6).

According to current data, B7H1 is commonly expressed in a range of human gastrointestinal malignancies, including pancreatic, gastric, esophageal, and colon cancers (7). By decreasing T-cell feedback, PD-L1 promotes immune evasion and stimulates tumor development (8). The PD-1/PD-L1 molecular pathway works as a cellular checkpoint to reduce inflammatory and immunological feedback against cancer. It does this by acting as a negative regulator of co-signaling pathways (9). T cells and tumor-infiltrating lymphocytes (TILs)’ function is inhibited by the interaction between PD-1 and PD-Ls, whilst immunosuppressive Tregs activity is increased (10). Because of this state, tumor cells hide from immune responses. By lowering the amount of Tregs and their suppressive activity while increasing the activity of effector T cells, blocking the PD-1/PD-L1 interaction improves antitumor immunity (11, 12).

**Figure 1:**
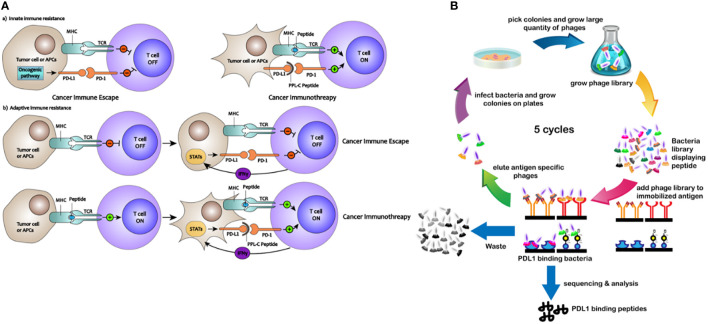
**(A)** Mechanisms of how PD-L1–binding peptides activate PD-L1–mediated inhibition of T cells. **(B)** Schematic description of the PD-L1–binding peptides by phage display library screening procedure.

A valuable technique for screening antibodies and improving their stability, specificity, and affinity is phage surface display (13). Utilizing fluorescently labeled reagents and flow cytometry, the ligand of interest is expressed on the surface of the phage, enabling quantitative measurement of certain binding peptide features, such as stability and affinity (14). Specific binding peptides present various advantages as drug candidates, including cost-effectiveness, improved stability, reduced immunogenicity, and enhanced penetration into organs or tumors (15, 16). For cancer detection and therapy, scientists have studied peptides that target cancer cells, cytokines, and membrane receptors (17). The processes by which PD-L1-binding peptides activate PD-L1-mediated suppression of T cells are shown schematically in **Figure 1A**. In this study, phage surface display techniques were employed to screen a specific and high-affinity PD-L1-targeting peptide. Overall, we demonstrate the potential for developing low-molecular-weight PD-L1-binding peptides as potent candidates for cancer immunotherapy.

## Materials and methods

### Reagents and materials

Human PD-L1 natural ORF mammalian expression plasmid (Cat: HG10084-UT), rabbit anti-human PD-1 antibody (Clone D67, Cat: 10377-RD67), recombinant human PD-L1 protein (Cat: 10084-H08H), human PD-L1/B7-H1/CD274 protein (Cat: 10084-HNAH), biotinylated mouse PD-1/PDCD1 protein (His tag) and recombinant human PD-1 protein (Cat: 10377-H08H) were purchased from Sino Biological Inc (Beijing). GM-CSF (Cat: 315-03) and Murine TNF-α (Cat: 315-01A) were purchased from PEPROTECH. The 12-mer peptide phage display library was supplied by the New England BioLabs, Ipswich, MA, Abundance: 10×10^9^. Lipofectamine 2000 was purchased from Invitrogen. Biotech Bioengineering (Shanghai) Co., Ltd provided most molecular reagents. Mitomycin C was obtained from Meilun Biotech. Endotoxin-free PD-L1 antibody (10F.9G2) was purchased from BioXcell. HRP-anti-M13 antibody (Abcam clone B62-FE2), HRP-anti-FITC antibody (Thermo Fisher Cat: PA1-86159), and FITC polyclonal rabbit antibody (Thermo Fisher Cat: 71-1900), goat anti-rabbit HRP-IgG antibody (Cell Signaling), and anti-human FITC-PD-L1 antibody (Abcam) were purchased from the given companies. The following antibodies from eBioscience, Miltenyi, BD Bioscience, BBILife Sciences, and Biolegend were used for flow cytometry: mouse CD4 (L3T4) MicroBeads, PE Rat IgG2b, κ Isotype Control, anti-CD3 (PE, clone 17A2), anti-CD4 (PE/Cy5, clone RM4-5; FITC, clone GK1.5), anti-CD8a, CD16/32 (TruStain fcX^TM^, clone 93), anti-CD49b (PE, clone HMα2), anti-Foxp3 (647, clone MF-14), biotin anti-mouse PD-L1 (Cat: 124305), PE streptavidin (Cat: 405203), CD3 monoclonal antibody (17A2), functional grade, eBioscience™, granzyme A antibody rabbit polyclonal to mouse 11288-1-AP (Proteintech). Anti-IFN-γ rabbit pAb (GB11107) and anti -CD8A Rabbit pAb (GB11068) were purchased from Servicebio. Nivolumab was gifted from Changhai Hospital in Shanghai.

### Animals and tumor cell lines

Female BALB/c (H-2d) mice, aged 4-5 weeks and weighing approximately 20-22g as well as Swiss ICR mice were purchased from Shanghai JieSiJie Laboratory Animal Technology (Shanghai, China). The mice were subcutaneously administered with free peptide or antibody drugs. All mice were treated according to the Guide for the Care and Use of Laboratory Animals of Fudan University. The investigators conducting the animal studies were not blinded. To prevent the liver and kidneys from overlapping, CT26 tumor cells were injected into the back of the mice. The mice were transplanted with 1×10^6^ tumor cells suspended in 200 μL saline, and this was injected into the subcutaneous area of the flank when the mice were between 4-5 weeks old. The animals were treated once their tumors reached an area of 25 mm^2^ (primary), 100 mm^2^ (medium), or 150 mm^2^ (advanced). The mice were then randomized into different treatment groups. For assessing the peptide's half-life in subcutaneous tissue, female Balb/c nude mice aged five to six weeks (SLAC laboratory animal, Shanghai) were used in this experiment. The half-life was calculated to evaluate the peptide dosing interval.

The FITC-conjugated PPL-C peptides were injected subcutaneously at 4 mg/kg. The corresponding FITC fluorescence intensity was measured using the Berthold LB983 at different intervals: 0, 5, 15, 30 min, 1, 2, 4, 6, 12, 24, 48, and 72 hours. Cell culture reagents were obtained from Gibco. The CHO cells, MDA-MB-231 (human breast cancer cell), MGC-803 (human gastric carcinoma cell), LLC (mouse lung cancer cell), and MC38 (mouse colon cancer cell) were acquired from the Laboratory of Molecular Immunology at Fudan University, Shanghai, China. CT26.wt cells were obtained from Naval Medical University’s immunization department. CHO-PDL1 cells were developed by transfecting CHO cells with a recombinant human PD-L1 full-length expression plasmid. In RPMI-1640 supplemented with 10% FBS and 100 U ml-1 penicillin/streptomycin, the CHO cell lines, MC38 and CT26.wt were kept alive. MDA-MB-231, MGC-803 and LLC were maintained by culture in DMEM with 10% FBS and 100 U ml-1 penicillin/streptomycin. The cell lines were grown at a temperature of 37°C with a 5% CO_2_ concentration in humidified incubators.

### Phage display peptide library biopanning

The filamentous phage M13KE 12-mer peptide phage display library underwent bio-panning following instructions from New England BioLabs, Ipswich, Massachusetts. To summerize the process in short, a total of 1.5 µg of recombinant human PD-L1 (Sino Biological Inc, 10084-H08H) was precisely absorbed onto a 96-well plate (Corning, NY) at 4 °C. After overnight incubation, the wells were blocked for 2 hours at 4 °C with a solution of 5% bovine serum albumin (BSA) in water. After thorough washing with TBST (0.5% Tween 20), 1×10^9^ phages were added to the PD-L1-coated wells and allowed to incubate for 2 hours at room temperature. After this stage, the nonspecifically or loosely cuvet phages were successfully removed by washing, while the high cuvet phages were eluted using a solution of 0.2 M glycine-HCl (pH 2.2) and 1 mg/mL BSA. Following their recovery, these phages were amplified in *E. coli* ER2738, titrated on IPTG/X-gal plates, and used for the next round of screening at a defined titer. The DNA was extracted and quantitated on an agarose gel following five rounds of bio-panning, compared to 0.5 g of pure single-stranded M13mp18 DNA (NEB 4040). The resulting DNA was then sequenced. From the phage display library, a selection of seven peptide fragments that displayed potential affinity for the PD-L1 protein was successfully identified and screened.

### Peptides synthesis

The peptide candidate which showed a strong affinity for the PD-L1 protein was selected for further analysis of its hydrophilicity. After modification, the peptide synthesis process was carried out using the standard FMOC method (Ningbo KangBei Biological Technology Co., Ltd). The N-terminus of the peptide was supplemented with amino acids, while the C-terminus was amidated and connected to fluorescein isothiocyanate (FITC) through the GGGSK- linker. To prolong the oligopeptide’s half-life, a commonly employed modification involving PEG (polyethylene glycol) was selected. Specifically, PEG8 (MW649) was attached to the C-terminal of the 12-mer peptide and connected to the side chain of amino acid K.

### Binding analysis of peptides and phages

After seven phages were grown and purified, the solution was diluted 10 times to produce eight gradients. To ascertain the phage’s affinity for PD-L1, an ELISA test was run. Briefly, 96-well plates were filled with 150 µL of PD-L1 (1 g/mL) in assay diluent (BioLegend, San Diego, CA), and incubated at 4°C overnight. 5% BSA in water was used to block the wells for two hours at 4°C. Following washing, 100 µL of phage diluent was added to each well, and they were shaken for two hours at room temperature. The wells were then treated with HRP-conjugated anti-M13 antibody at dose of 1:5000. With 100 µL of HRP substrate, the reaction was observed, and the OD_410_ was determined. By deducting the OD value of each phage binding to BSA-coated wells from the OD value of that phage binding to PD-L1, the specific binding of each phage to PD-L1 was computed. Three duplicate runs of this assay were made. GraphPad Prism 6 software was used to compute each phage binding affinity by nonlinear regression and convert it to a Scatchard plot. ELISA was used to evaluate the candidate peptides capacity to inhibit PD1/PD-L1 binding. The subsequent stages were the same as those previously outlined. Following incubation, OD_410_ was determined by goat anti-rabbit monoclonal antibody that had been HRP-labeled and rabbit anti-human PD-L1 protein monoclonal antibody.

### Estimating the *K_D_* value of PPL-C to PD-L1

The binding affinity of the peptide to the PD-L1 protein was determined by measuring the dissociation constant (KD). FITC was incorporated into multiple peptide sequences, which were then tested using HRP-labeled anti-FITC antibodies. When the concentration of PD-L1 protein remained constant, the concentration of saturated peptides varied. The level of saturation indicates the affinity between the peptides and PD-L1. To investigate the epitope binding sites on PD-L1, a competition ELISA was conducted using the aforementioned method. It is imparative to note that three repetitions are required for all experiments.

### Assessment of the soluble peptides binding specificity

Fluorescence-based ELISA and cell-based tests were used to examine the binding specificity of PPL-C to PD-L1. To perform the ELISA, fluorescent plates were coated with 100 μL human PD-L1 (hPD-L1), mouse PD-L1 (mPD-L1), and BSA in PBS (pH 7.4) overnight at 4°C, respectively. Blocked the plates with 1% BSA (in PBST) for an hour at room temperature and rinsed twice with PBST. Then added peptides (20 μmol/L FITC-labeled PPL-C/Control peptide) and incubated for 1.5 hours at room temperature. With excitation at 488 nm and emission at 515 nm, the Cytation 3 instrument (BioTek) was used to measure the relative fluorescence intensity. Both attached and detached cells were used in the cell-based flow cytometry analysis. With 0.25% trypsin, cells from the following types were detached: CHO, CHO-PD-L1 (CHO cells transfected with a recombinant human PD-L1 full-length expression plasmid), MDA-MB-231, MC38, CT26, and LLC cells. After that, cells were treated for 40 minutes at 4°C on an inversion shaker with PD-L1 antibody from Biolegend and 4 μmol/L FITC-labeled peptides (PPL-C/Control peptide). Each tube was washed twice with PBS following incubation and flow cytometry was carried out using a FACSort device. FlowJo software was used to evaluate the data that was acquired.

### Ligand inhibition assay

To assess the effect of PPL-C peptide interference, PD-L1 binding to PD-1 was investigated using ELISA and cell-based analysis. For the ELISA, 100 μL of mouse PD-L1 in PBS was coated in each well of a 96-well plate and incubated the plate at 4°C overnight. On the second day, 1% BSA in PBST was used to block the plate. Each well was then filled with 200 nmol/L biotinylated PD-1 and FITC-labeled PPL-C peptides (at final concentrations of 40, 20, 10, 5, or 2.5 μmol/L) before 1.5 hours incubating at room temperature. After two PBST washes, the relative FITC fluorescence intensity was measured using a Cytation 3 instrument. SAPE was applied to the wells and incubated for 30 minutes at room temperature to identify biotinylated PD-1. After two PBST washes, the Cytation 3 device was used to detect PE fluorescence intensity. MGC-803, CT26, and LLC cells were dissociated with 0.25% trypsin and diluted to a final concentration of 1×10^6^ cells/mL for the cell-based study. The cells were then treated with FITC-labeled PPL-C peptide for 60 minutes at 4°C on an inversion shaker in the presence of 200 nmol/L biotinylated PD-1. The final FITC-labeled PPL-C peptide doses used were 20, 8.0, 4.0, 2.0, 0.8, 0.4, or 0 mol/L. SAPE was added after two PBS washes, and the mixture was incubated for an additional 30 minutes. Samples were analyzed by flow cytometry.

### Proliferation assay

To observe the impact of PPL-C on tumor cells and lymphocyte proliferation, the Counting Kit 8 (CCK8) reagent was employed. 20,000 cells/well were cultured in 96-well plates. Cells were treated for 8 or 24 hours with PPL-C (0.1, 0.25, 0.5, and 1 mg/ml) or not. Following the manufacturer guidelines, 10 µl of the CCK8 reagent (from Biotech Bioengineering, Cat: E606335) was applied to each well two hours before the incubation period ended. Using an ELISA plate reader from Molecular Devices (United States) the sample absorbance was calculated at 450 nm. To ensure repeatability, each experiment was replicated. By assessing absorbance at 450 nm, the CCK8 assay measures cell viability and proliferation.

### T-cell activation assay

Following the manufacturer’s instructions, mouse peripheral blood mononuclear cells (PBMCs) were isolated from a healthy mouse using separation fluid. Then, CD4 (L3T4) MicroBeads were used to separate CD4+ T cells from PBMCs, and a CD4-PE/Cy5 antibody was employed to determine effectiveness. Cells were adjusted to a concentration of 5×10^5^ cells/mL in IMDM supplemented with 10% FBS and 20 ng/mL IL-2 to produce CD4+ T-cell blasts. A 1:1 ratio of mouse T-activator CD3/CD28 beads was introduced to the cells. Every other day, new media was added, and on day 7, cells were collected and stored for later use. 100 μL of anti-mouse CD3 (1 μg/mL) were applied to each well and incubated at 4°C overnight. Next, the wells were rinsed twice with 200 mL of PBS. The wells were then filled with 100 μL of PD-L1 (10 g/mL) and the plates were incubated at 37°C for 3 hours before receiving two PBS washes. Then, varying quantities of PD-L1 monoclonal antibodies and peptides (PPL-C or control peptide) were added to the appropriate wells, and the plates were incubated at 37°C for an hour. The wells were then washed twice with PBS. Finally, CD4^+^ T cells (5×10^4^ cells/well) were added, then centrifugated the plate at 1,000 RMP for 2 min. The cells were then cultured for three days at 37°C in a humidified incubator with 5% CO_2_. A mouse IFN-γ ELISA Kit was used to assess the quantity of IFN-γ in culture supernatant. The Cell Counting Kit-8, which as stated in the reagent instructions, shows a more linear connection between cell number and OD_450_ value. It was used to measure T-cell proliferation.

### Mixed lymphocyte reaction

Fresh PBMCs were cultured in IMDM at 37°C for 2 hours and washed twice in PBS. Then cells were cultured in IMDM supplemented with 10% FBS, 25 ng/mL GM-CSF, and 50 ng/mL IL-4. Fresh medium was added every three days. TNF*-*α (50 ng/mL) was added on the sixth day to stimulate the maturation of dendritic cells (DCs) for 24 hours. The mature DCs were then frozen to be harvested later. Solid tumor tissues from the CT26 tumor-bearing animal have been removed and fragmented to collect tumor cells. In RPMI 1640 medium supplemented with 10% FBS, 2 mM L-glutamine, 200 mg/mL streptomycin, and 200 U/mL penicillin, these fragments were subjected to digestion with a combination of triple enzymes (collagenase, DNase, and hyaluronidase) for a maximum of 2 hours. The resultant cell suspension was then grown in complete RPMI 1640 medium at 37°C with 5% CO_2_ for 24 hours. The goal of this culture phase was to re-establish PD-L1 expression on the tumor cell surface since collagenase digestion may have destroyed it. DCs and tumor cells were both given a 2-hour treatment with mitomycin C (MMC) at a dosage of 100 μg/mL before the start of mixed lymphocyte responses (MLR). After thawing, the CD4+ T cells were cultured for overnight. In a total volume of 200 μL of IMDM supplemented with 10% FBS, 1×10^5^ cells were sown in each well with a ratio of 1:5:10 for DCs, CT26 tumor cells, and T cells, respectively. Cell growth and IFN-γ levels in culture supernatants were measured on day 3.

### Bioinformatics analysis

To construct the polypeptides and obtain their initial structures, we applied the Insight II Building and Edit Protein module. The CHARMM force field was employed to minimize energy and molecular dynamics models. The PD-L1 receptor and 12 peptide binding sites were based on PDB crystal structures 3BIK, 4ZQK, and 5J89. Both receptors and peptides were subjected to the charmm27 force field. We used the Zdock module in the Insight II business package to dock the 12 peptides and PD-L1 protein, based on the binding site set. The rDock module was then used for optimization. Finally, the docked protein models were analyzed by the Analysis Docked Protein module.

### Evaluation of tumor volume, growth and survival curves

The mice’s tumor volumes were measured by calipers after they were randomly allocated to various treatment groups. Every day tumor volumes were measured along axes (a and b) that are orthogonal to one another, and they were estimated by the equation tumor volume = π/6 × a × b^2. After the tumor was established (approximately 7 days), each group was subcutaneously injected with PPL-C (6 or 11 mg/kg), control peptide (11 mg/kg), endotoxin-free PD-L1 antibody (200 μg), or control antibody, following specific treatment procedures outlined in the flow chart. The Kaplan-Meier technique was used to determine survival differences for each group, and the log-rank test using the survival R package version 2.38-3 was applied to get the overall p-value.

### Enzyme-linked immunosorbent assay

After 14 days of continuous administration, 0.5-1.0 ml of peripheral blood was collected from the mice. Serum was isolated from peripheral blood using the posterior orbital venous plexus blood collection method. The serum was obtained by centrifuging at 2000 RMP for 10 minutes. Mouse total lymphocytes were isolated from the spleen using Ficoll density gradient fractionation (Shanghai Biotech Bioengineering). In 200 μL of RIPM medium with 10% FBS, 1×10^6^ tumor cells were seeded in each well with a CT26: lymphocyte ratio of 1:2 and incubated for 48 and 72 hours. Cell growth medium was harvested by centrifuging at 2000 RPM for 10 min. For the analysis of cytokine production, lymphocyte growth media served as a positive control and cell-free growth medium served as a negative control. Following the manufacturer’s instructions, the levels of IL-2, IL-4, IL-6, IL-10, TNF-α and IFN-γ in the blood and cell growth media were assessed using LEGEND MAX™ ELISA kit (BioLegend Inc, USA). Three duplicates of each experiment were carried out.

### Cytotoxicity and reactivation of mouse lymphocytes after PPL-C treatment

After 14 consecutive days of administration, total lymphocytes were isolated from mouse spleens using Ficoll density gradient fractionation, which was provided by Shanghai Biotech Bioengineering. Before co-incubation with lymphocytes, CT26 cells were treated with 100 μg/mL mitomycin C (MMC) for 2 hours for reactivation experiments. For cytotoxicity experiments, about 1×10^6^ tumor cells were seeded in each well with a CT26: lymphocyte ratio of 1: 2 in 200 μL RIPM medium plus 10% FBS for 48 h. A corresponding negative control group was also designed. During the final two hours of the incubation, 10 µL of CCK8 reagent was added to each well following the manufacturer’s instructions (Biotech Bioengineering Cat: E606335). Using an ELISA plate reader (Molecular Devices, USA) the absorbance was determined at 450 nm. There were three duplicates of each experiment.

### Flow cytometric analysis

To differentiate lymphocytes, spleen monocytes were separated via isodensity centrifugation. For flow cytometry evaluation, cell-surface and intracellular staining was achieved through reagents from BD Biosciences or Biolegend, following previously described protocols. Re-suspending the ***1×*** 10^6^ cells in 100 μL of PBS before incubating them with the appropriate antibodies. Data were collected using a BD Biosciences FACSCalibur flow cytometer, Germany, and analyzed using FlowJo 7.6 software, USA. Based on scatter signals and 7-Amino-actinomycin D fluorescence, dead cells, and cell debris were removed from the study.

### Immunofluorescence

Cells were cultured on microscope slides and washed three times in PBS for 15 minutes each. Then fixed the cells with 4% paraformaldehyde at room temperature for 15 minutes. After that, cells were treated with FITC-conjugated PPL-C or a control solution at room temperature for 2 hours. Following the treatment, the cells underwent three additional washes in PBS, each lasting 30 minutes. Images of the cells were captured using a wide-field fluorescence microscope (Zeiss in Jena, Germany), following an additional cleaning procedure.

### Immunohistochemistry

On day 15, all mice were euthanized, and tumor tissues were collected. Immunohistochemistry (IHC) was used to identify the expression of mouse IFN-γ, CD8, and GZMB in the spleen or tumor tissues of each group with the relevant antibodies. Four normal mouse tissues were used to assess the target specificity of FITC PPL-C and FITC-control peptides.

### Statistical analysis

The analysis of differences between treatment groups was conducted through GraphPad Prism 6.0 software. Statistical techniques such as the 2-tailed unpaired t-test or other appropriate methods described in the article were employed. The data are presented as mean ± SEM. Survival analysis was conducted using the Kaplan-Meier test. The differences between survival curves were assessed using the log-rank test. A significance level of P < 0.05 was employed to determine statistical significance in all cases.

## Results

### Identification of PD-L1-binding peptides with high affinity and specificity from a phage display library

To identify peptides that bind to PD-L1, an NEB dodecapeptide phage surface display library with approximately 9×10^7^ clones on LB agar plates was utilized. The exogenous random polypeptide gene was genetically engineered to be transferred into the interior of the M13 phage, which was then expressed on the surface of the phage (screening procedures are shown in **Figure 1B**). Polypeptides with an affinity for the target protein were selected via bio-panning, and after three to five rounds, the highest frequency clone was obtained (**Supplementary Figure S1**). Seven different peptide sequences were identified (**Supplementary Table S1**) with no obvious consensus sequence. The binding properties of these seven different peptides displayed on bacteria surfaces were initially analyzed using ELISA. All saturation curve results demonstrated that clone PL-8, clone PL-20, and clone PPL-C exhibited a higher binding affinity and specificity than any other clones, with no significant differences between thems (**Supplementary Figure S2A**).

### PPL-C binding specificity to PD-L1 and blockade PD-1/PD-L1 interaction

Candidate peptides with significant affinity for the PD-L1 protein were selected and analyzed for hydrophilicity and synthesized for further experiments after modification (**Supplementary Table S2**). Fluorescent group FITC was added to the C-terminus of several synthesized peptide sequences, which allowed detection using an HRP-labeled anti-FITC antibody. When the PD-L1 protein concentration was held constant, the concentration of saturated peptides differed, indicating the affinity of the selected peptides for PD-L1. Our preliminary data showed that all three candidate peptides were compatible with PD-L1 protein. The KD values were 0.75 μM for PPL-C, 0.32 μM for PL-8, and 4.30 μM for PL-20 (**Supplementary Figure S2B**). An ELISA assay for the competition of the three candidate peptides for PD-L1 affinity sites indicated that only PPL-C competed with PL-20, suggesting that the binding sites may coincide (**Supplementary Figures S3E, F**). To determine whether the three candidate peptides were antagonistic, we conducted ELISA experiments to investigate whether PPL-C, PL-8, and PL-20 could compete with PD-1 to bind PD-L1. The data showed that PPL-C had a significant blocking effect on the PD-L1/PD-1 pathway and that the inhibitory effect increased as the peptide concentration increased. PL-8 and PL-20 blocked moderately (**Supplementary Figure S4**). As a result, clone PPL-C was selected for further experiments and designated PPL-C.

**Figure 2:**
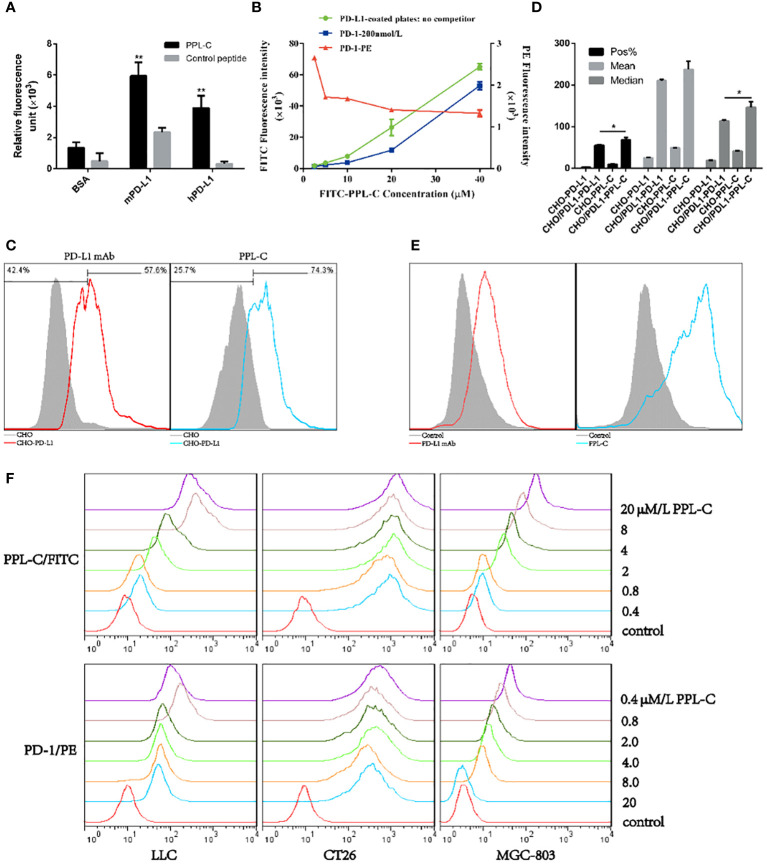
The physicochemical properties of PPL-C peptides. PPL-C binding specificity to hPD-L1, mPD-L1, and BSA was analyzed by fluorescence-based ELISA (**A**, n = 3). Ligand inhibition assay analysis of the changes in fluorescence signals of PPL-C-FITC (green and blue lines) and PD-1-PE (red lines) with the increased concentration of peptide was analyzed by fluorescence-based ELISA (**B**, n = 3). The binding ability of PPL-C (blue lines) or PD-L1 mAb (red lines) to CHO (gray) and CHO-PD-L1 cells was determined by flow cytometry (**C**, n = 3). Statistical analysis of the results of three repeated flow experiments, where Pos% is the fluorescence positive rate, and Mean and Median are the mean fluorescence intensity values and median fluorescence intensity values (**D**, n = 3). PD-L1 expression on CT26 was detected by flow cytometry **(E)**. Different concentrations of PPL-C/FITC peptides and a single concentration of biotinylated PD-1 were incubated simultaneously to detect PD-1 binding capacity on LLC, CT26 and MGC-803 cell lines **(F)**. Data, mean ± SEM; *,P < 0.05; **,P < 0.01.

To confirm whether peptide PPL-C is specifically bound to PD-L1, we used both ELISA and flow cytometry. A scrambled control peptide was used in the ELISA experiments (**Supplementary Figure S5**). PPL-C showed a strong affinity for PD-L1 protein in fluorescence-based ELISA (**Figure 2A**). The results indicated that PPL-C displayed cross-reactivity to both mPD-L1 and hPD-L1, but displayed higher specificity to mPD-L1. The flow cytometry analysis showed that PPL-C could significantly bind to cell-surface-expressed PD-L1 in CHO-PD-L1 cells (**Figure 2C**) and CT26 cells (**Figure 2E**), compared to PD-L1-negative cells. Interestingly, PPL-C bound to PD-L1 more effectively than a PD-L1 monoclonal antibody (mAb) (**Figure 2D**). Antibody drugs targeting the PD-1/PD-L1 signaling pathway have proven effective in clinical treatment. However, these antibodies, due to their large molecular weight and potent immunogenicity, pose risks of toxic side effects such as anti-antibody production, ADCC effect, and inflammation. In contrast, PPL-C, as a small molecule peptide, possesses a diminutive molecular weight, heightened stability, reduced immunogenicity, and lower toxicity, rendering it a safer alternative to PD-L1 antibodies. To further investigate the PPL-C binding specificity in a more physiological environment, we evaluated its ability to bind to MDA-MB-231, MC38, and LLC cells using flow cytometry. PD-L1 expression was measured in these cells, with MDA-MB-231 and LLC cell lines showing higher PD-L1 expression than MC38. PPL-C demonstrated higher affinity to MDA-MB-231 and LLC cells, while it had low affinity for MC38 cells which express relatively low levels of PD-L1 (**Supplementary Figure S6**). To validate our results, we performed immunofluorescence analysis, which demonstrated that FITC-PPL-C effectively binds to the cell membrane in LLC cells (**Supplementary Figure S7**). By scrutinizing the alterations in splenic NK cells post-immunotherapy with PD-L1 antibodies and PPL-C, we assessed the comparative biosafety of PPL-C versus PD-L1 antibodies in treatment. In comparison with the Control peptide (10.80%), the PD-L1 antibody treatment group exhibited a fourfold reduction in the percentage of NK cells (2.70%), while no significant reduction was observed in the PPL-C treatment group (7.79%) (data not shown). The subcutaneous half-life in nude mice and plasma concentration half-life in rats of PEG8-modified FITC-PPL-C approximate 10 hours and 4 hours, respectively. Immunohistochemistry results also corroborate that PPL-C remains sparingly enriched in normal tissues (**Supplementary Figure S8**), underscoring its safety profile as a small molecule PD-L1/PD-1 blocker.

These results confirm that PPL-C can specifically bind not only to free PD-L1 but also to cells that express PD-L1 on cell membranes. We used ELISA and cell-based analysis to examine how PPL-C peptides affected PD-L1 binding to PD-1 in the ligand inhibition experiment. According to the ELISA results, PPL-C could successfully bind to PD-L1 even when PD-1 was present (**Figure 2B**). Additionally, as the PPL-C peptide concentration was raised, the fluorescence signals of PD-1-PE decreased. According to the flow cytometry data, PD-1 ability to bind to LLC, CT26, and MGC-803 cell lines was reduced as the PPL-C concentration was raised (**Figure 2F**).

### PPL-C reverses PD-L1-mediated suppression of T-cell activation

**Figure 3:**
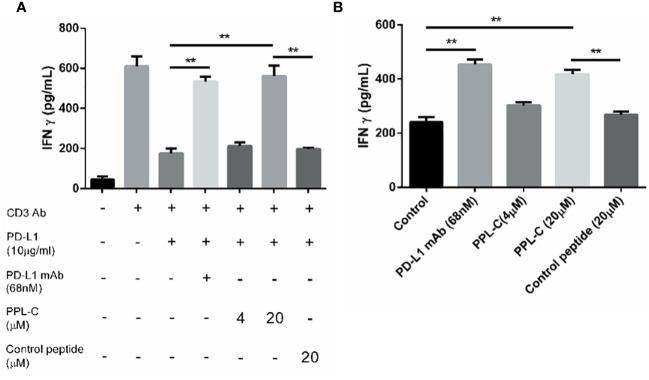
PPL-C reactivated T-cell function was verified by T-cell activation assays (**A**; n = 3) and MLR assay (**B**; n = 3). T-cell activation was positively correlated with IFN-γ production. Data, mean ± SEM; *,P < 0.05; **,P < 0.01.

To examine PPL-C potential to inhibit PD-L1 and activate CD4+ T lymphocytes, CD4+ T cells were isolated from PBMCs using CD4 (L3T4) microBeads. Subsequently, these isolated CD4+ T cells were stimulated with anti-CD3 and anti-CD28 antibodies in the presence of PPL-C, PD-L1 monoclonal antibody (mAb), or a control substance. IFN-γ production and cell proliferation levels were measured. PPL-C-treated CD4+ T lymphocytes showed a significant increase in IFN-γ release compared to control peptide-treated CD4+ T lymphocytes (**Figure 3A**). Similarly, at a PPL-C concentration of 20 μmol/L, T-cell proliferation increased significantly (**Supplementary Figure S9C**). These findings support prior studies showing that anti-PD-L1 antagonists may successfully activate CD4+ or CD8+ T cells to release IFN-γ (18, 19). As a result, PPL-C stimulates T cell activation and IFN-γ secretion. To assess the PPL-C efficacy in a more realistic setting, a mixed lymphocyte response (MLR) was conducted. In MLR, lymphocytes will activate and proliferate after being stimulated by allogeneic antigens and produce a wide variety of cytokines to promote the differentiation of natural killer cells (NK), lymphokine-activated killer cells (LAK), and cytotoxic T lymphocytes. This involved combining mature DCs with CD4+ T cells and tumor cells obtained from different donors to induce allogeneic T cell activation. To stimulate CD4+ T cells, IL-2 and CD3/CD28 antibodies were utilized. This experimental setup aimed to verify the effectiveness of PPL-C in a context that closely resembles the natural immune response. 20 μmol/L PPL-C demonstrated a significantly higher release of IFN-γ compared to the control and control peptides (**Figure 3B**), while all groups showed similar proliferation outcomes (**Supplementary Figure S9D**). It is significant to note that the amount of IFN**-**γ produced during PBMC activation was substantially higher than that seen during MLR experiments. The PBMC test and MLR study are completely separate systems with distinct immune activation processes and activation statuses, which may explain this discrepancy. The first signal (anti-CD3 antibody) and the second signal (anti-CD28 antibody) activate T cells in the PBMC system, whereas allogeneic antigens from DCs trigger antigen-specific responses in the MLR system. Therefore, the MLR system activation levels were much lower (20). Additionally, PPL-C cytotoxicity towards CT26 tumor cells was also examined. Following PPL-C administration, CT26 cells grew normally (**Supplementary Figures S9A, B**), indicating that PPL-C does not directly kill tumor cells. Based on these results, it is hypothesized that PPLC may function by activating anti-tumor immunity rather than directly killing CT26 tumor cells (12).

### Superior antitumor immunity and efficacy of PPL-C

**Figure 4:**
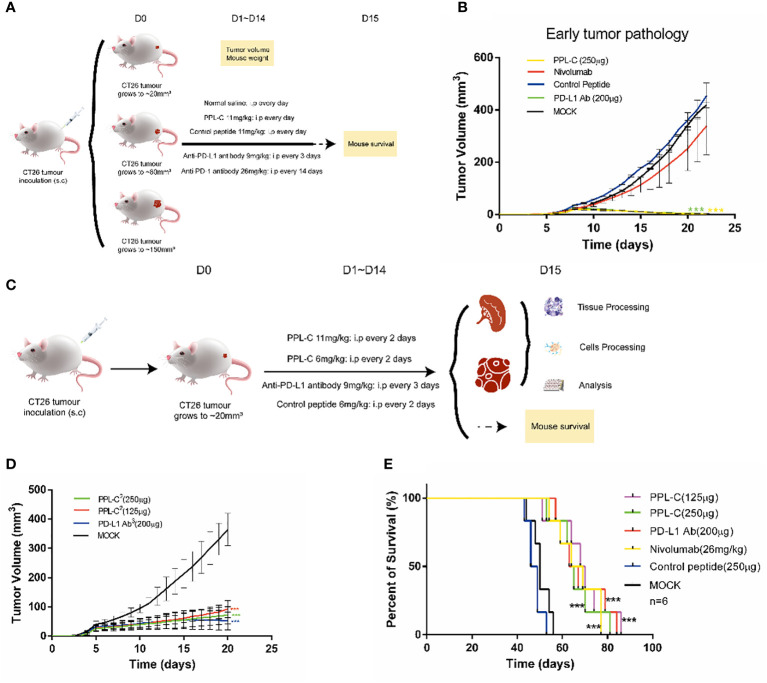
PPL-C inhibited tumor growth in a transplantation tumor. Treatment schedule for mice bearing subcutaneous CT26 tumour of different tumor stages for the immunotherapy **(A)** and treatment schedule for mice growing to 20 mm^3^ for immunotherapy **(C)**. Growth inhibition of early tumor pathology via PPL-C (**B, D**, n = 3). Survival prolonging curves for CT26-tumor-bearing Balb/c mice treated with various treatment methods (**E**, n = 6). Data, mean ± SEM; ***, P < 0.001.

To assess the therapeutic efficacy of the peptide *in vivo*, tumor growth and survival experiments were conducted in mice employing PPL-C (**Supplementary Table S3**). A total of sixty Balb/c mice were subcutaneously injected with either 1×10^5^, 5×10^5,^or 1×10^6^ CT26 cells on the right flank. CT-26 is a murine colorectal cancer derived from BALB/c mice. The syngeneic tumor model is one of the most commonly used mouse solid tumor models and is highly immunogenic. Mutation patterns of onco-relevant genes, gene expression signatures, and regulatory pathways in CT26 cells are consistent with their colonic epithelial origin and share features with human primary colorectal cancer (CRC). These mutations and expression profiles are similar to those reported in sporadic, undifferentiated, treatment-refractory, metastatic human colorectal cancer. After five or seven days, when the tumor volume reached either 20 mm^3^ (early tumor stage), 100 mm^3^ (medium tumor stage), or 150 mm^3^ (advanced tumor stage), the mice were randomly divided into five groups, each consisting of six mice. This experimental design allowed for the evaluation of PPL-C effects on tumor growth and overall survival in a controlled manner. Various administration methods of PPL-C (11 mg/kg) were employed, which included subcutaneous injection around the tumor once every day, every two days, every seven days, or every thirteen days for fourteen days. Normal saline was injected as a negative control. The therapy procedures are illustrated in **Supplementary Figure S11A**. Results are presented in **Supplementary Figures S11B**, **C** illustrate the significant inhibition of implanted CT26 tumor growth in Balb/c mice when treated with PPL-C. This effect was observed in both the daily and every two-day injection models, with a statistical significance of P < 0.01 and P < 0.001 compared to the negative control, respectively. Additionally, no significant loss in body weight was observed during the treatment. The survival of mice in each group was monitored daily until all mice had succumbed to the tumor. The data revealed that PPL-C treatment resulted in a nearly 50% increase in the survival time of CT26 tumor-bearing mice, suggesting its potential as a therapeutic agent for prolonging survival in this context (**Supplementary Figure S11D**). Considering the half-life of the peptide in subcutaneous tissue (**Supplementary Figure S10**), peptides were administered every day or every other day. To further confirm the inhibitory effects of PPL-C on tumor growth, 90 Balb/c mice were subcutaneously injected with 1×10^5^, 5 ×10^5^ or 1×10^6^ CT26 cells on their right flank. After five or seven days(As shown in **Figures 4A, C**, **Supplementary Figure S11A**, it is defined as D0), when the tumor volume reached 20 mm^3^, 100 mm^3^, or 150 mm^3^, the mice were randomly divided into five groups, each comprising six mice. The therapy procedures are shown in **Figure 4A**. Separate injections of normal saline and Anti-PD-1 antibody (Nivolumab) were used as controls. As the tumor stage shortened, both PPL-C and Anti-PD-L1 antibody treatments significantly inhibited tumor growth compared to the animals in the control group (with P < 0.01 and P < 0.001 compared to the negative control, respectively). Surprisingly, it was even found that the tumor completely disappeared at the early stage of tumor pathology (**Figure 4B**, **Supplementary Figures S11E, F**). After optimizing the treatment, the dose of PPL-C was reduced and administered subcutaneously around the tumor once every day or every other day (**Figure 4C**). The treatment with PPL-C using both methods demonstrated a significant inhibition in the growth of CT26 tumors, as indicated by **Figure 4D**, **Supplementary Figures S11G**, **H**. Furthermore, the administration of PPL-C resulted in a substantial increase in the survival time of CT26 tumor-bearing mice, extending it by approximately 50% compared to the control peptide (81 d *v.s*. 53 d, P<0.001). These findings suggest the potential of PPL-C as an effective therapeutic agent for inhibiting tumor growth and improving survival outcomes in CT26 tumor-bearing mice (**Figure 4E**). Moreover, safety tests were conducted in five groups of Swiss mice by twice intraperitoneal injection with 30 times effective amounts (180 mg/kg) of the test compounds, with normal saline as a control. Signs of toxic effects were observed during the first 72 hours, and observations were continued for 14 days on the surviving mice. No significant changes were observed in physiological and behavioral status, weight gain, blood biochemistry, or organ weight. Overall, no acute toxicity of PPL-C was observed (data not shown).

### Lymphocytes of CT26 tumor-bearing mice counteract Tregs differentiation via a T-cell-dependent mechanism after PPL-C treatment

**Figure 5:**
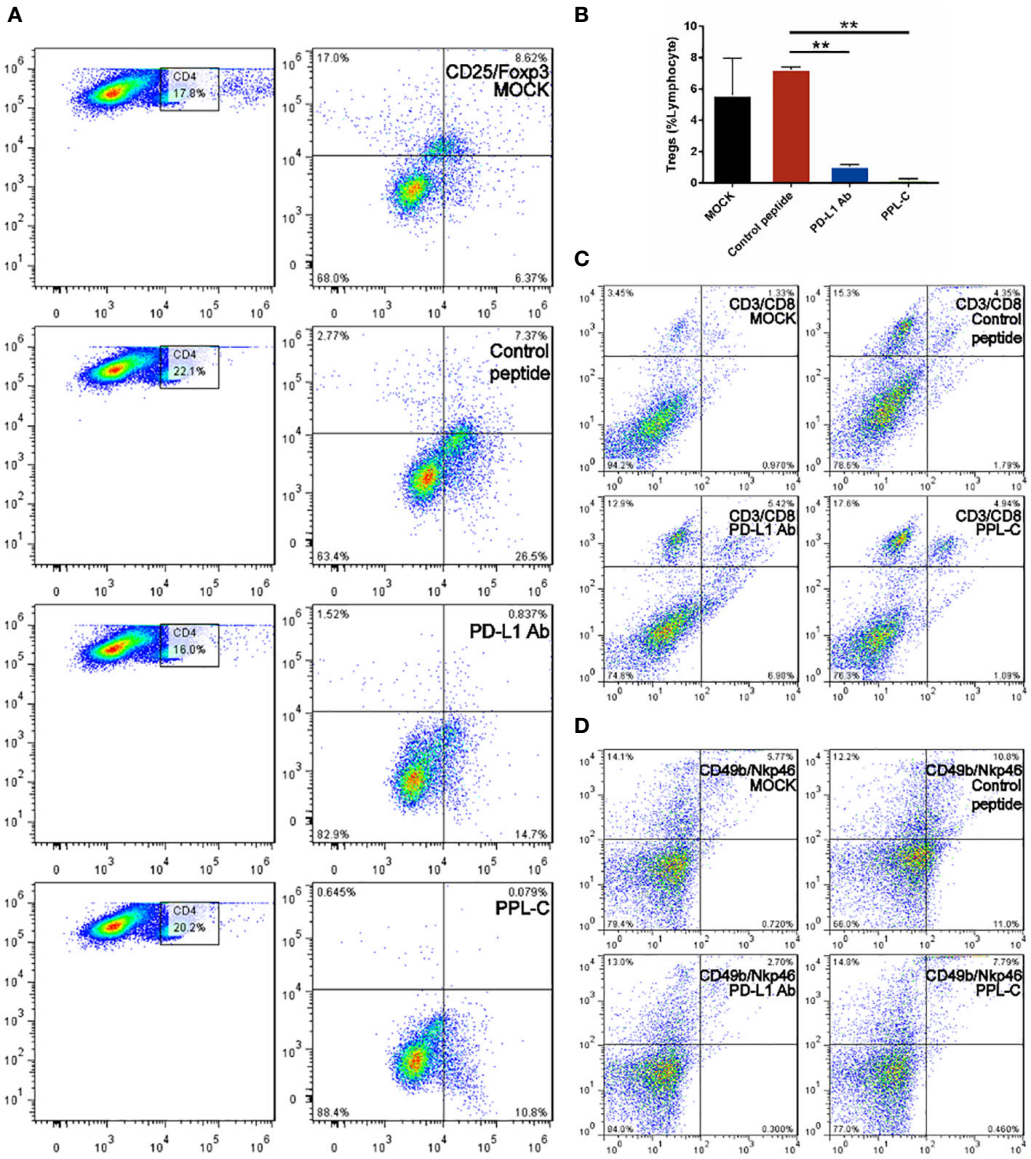
FACS analysis of the spleen from a Balb/c mouse 14 days after challenge with various treatments. Flow cytometric analysis of the comparative *in vivo* effect of PPL-C and PD-L1 on regulatory cells (Tregs, CD4+CD25+Foxp3+) in CT26-bearing mice **(A, B)**. The representative plots show the percentages of total splenic lymphocytes in CD3+CD8+ (cytotoxic T cell, **C**) and CD49+NKp46+ (NKT, **D**). Numbers in the corners indicate the percentage of cells in the gate. The data in **(B)** are shown as mean ± SEM, **, P < 0.01 (n = 3).

**Figure 6:**
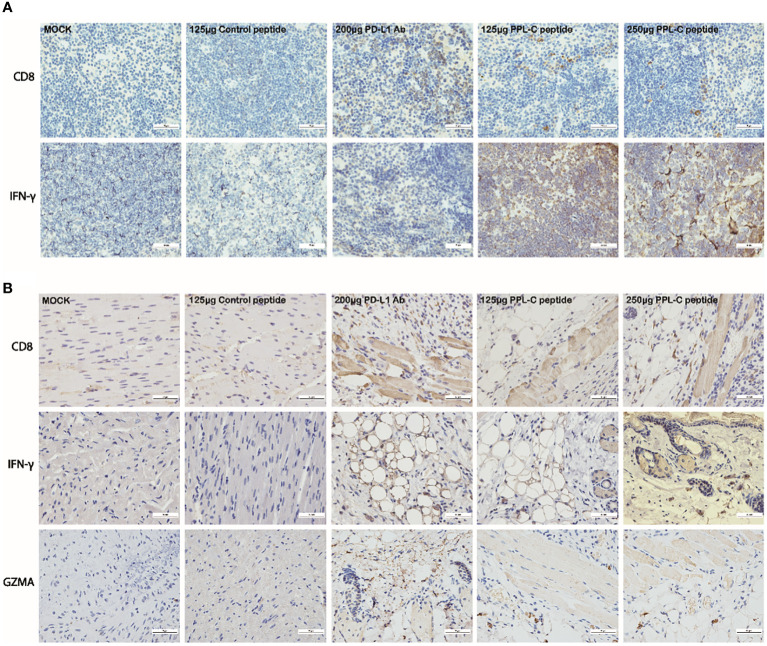
Representative images of CD8 and IFN-γ immunostaining on spleen tissues for each group **(A)**. Representative images of CD8, IFN-γ and GZMA immunostaining on tumor tissues for each group **(B)**. Scale bars, 50 μm. Magnification: 40×.

Additionally, PPL-C showed no discernible effect on newly separated PBMC cells without anti-CD3 (**Figure 3A**). This suggests that T cells, not NK cells, are responsible for the IFN-γ upregulation brought on by PPL-C. During tumor immunity, Tregs suppress excessive immune responses and promote immune escape of tumor cells by expressing inhibitory factors such as CTLA4, IL-10, and TGF-β. Additionally, Tregs can continuously infiltrate the tumor microenvironment (TME) through chemokines and participate in suppressing anti-tumor immune responses. Flow cytometry was used to determine wheter Tregs (CD3+CD4+CD25+Foxp3+) were present in the spleen since Tregs block cytotoxic T lymphocytes (CTLs) from mounting effictive antitumor immune responses (21). **Figures 5A**, **B** demonstrate that the frequency of Tregs in the immunotherapy groups (such as PPL-C or PD-L1 Ab) was significantly lower than the control peptide or PBS group. This demonstrates that PPL-C greatly reduces tumor immunological tolerance. CD8+ CTL proliferation was assessed by flow cytometry to further illustrate the PPL-C immunotherapy-induced antitumor immune response. In comparison to the untreated control group, PPL-C dramatically increased the proliferation of tumor-infiltrating CTLs (CD3+/CD8a+), as seen in **Figure 5C**. As PPL-C effectively stimulated T cell activation, we examined the proliferation of NK T cells by flow cytometric analysis but found no significant difference compared with the control group (**Figure 5D**). We observed the expression of CD8, IFN-γ and GZMA in both tumor and spleen tissues to further demonstrate that PPL-C inhibitory effects on tumor development were mediated by T-cell reactivation. IFN-γ and GZMA are two cytokines that are released into the tumor microenvironment (TME) when T cells are stimulated, which starts the tumor cell-killing process. IFN-γ and GZMA expression in tumor tissues significantly increased in the PD-L1 Ab and PPL-C groups compared to the untreated and control peptide groups, according to immunohistochemistry (IHC) analysis findings (**Figure 6B**). These findings show that our peptide prevented PD-1 and PD-L1 interaction to decrease tumor growth and activate T cells. The IHC results (**Figure 6A**) also indicated a significant increase in CD8 and IFN-γ expression in spleen tissues in the PD-L1 Ab and PPL-C groups, which is consistent with the proliferation of CD8+ CTLs observed through flow cytometric analysis (**Figure 5C**).

### Lymphocytes of CT26 tumor-bearing mice showed immune reactivation and cytotoxicity via upregulation of IFN-γ and downregulation of IL-10 cytokine secretion after PPL-C treatment

**Figure 7:**
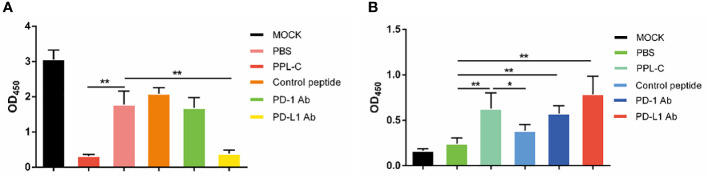
Lymphocyte cytotoxicity (**A**, n = 6) and proliferation (**B**, n = 6) analysis of spleen from a CT26-tumor-bearing mouse 14 days after challenge with various treatments. Data, mean ± SEM; *, P < 0.05; **, P < 0.01.

**Figure 8:**
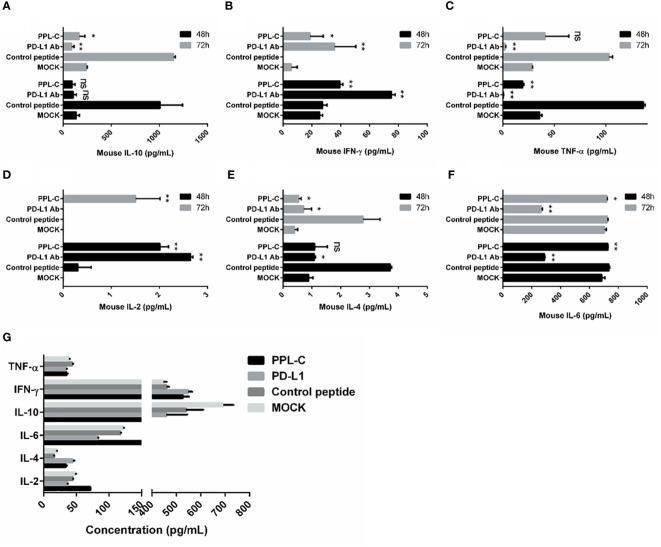
Splenocytes from various treatments were co-cultured with CT26 tumor cells *in vitro* for 48 and 72 hours. Supernatants were collected and IFN-γ was measured by an ELISA assay **(A–F)**. ELISA assay analysis of serum from a CT26 tumor-bearing mouse 14 days after challenge with various treatments **(G)**. This experiment was repeated twice with similar results. Data, mean ± SEM; *, P < 0.05; **, P < 0.01.

In many types of tumors, PD-L1 is abnormally highly expressed on the surface of tumor cells and binds to PD-1 on the surface of tumor-infiltrating T lymphocytes, thereby inhibiting the normal activation of T cells. The PD-1/PD-L1 signaling pathway inhibits the immune killing effect of T lymphocytes through multiple mechanisms. Phosphorylated Akt, mTOR, S6, ERK2, and other molecules are important for Tregs growth and differentiation. PD-L1 promotes the differentiation of Tregs by up-regulating the secretion of these molecules, thereby limiting the function of T cells. To confirm whether lymphocytes in immunotherapy-treated mice have been activated, T lymphocyte reactivation and T cytotoxicity are observed after lymphocytes co-incubate with tumor cells. As shown in **Figure 7**, lymphocytes from PPL-C treated mice significantly inhibited tumor cell proliferation and notably promoted reactivation, when compared to untreated and control peptide groups. By assessing the blood concentration of pro-inflammatory mediators and cytokines, we assessed the infiltration of CD8+ T cells and the mitigation of Treg differentiation-induced systemic immune responses. Compared to the untreated and control peptide groups, PPL-C significantly promoted IFN-γ, IL-2, IL-4 and IL-6 secretion in the immunotherapy group, while lower IL-10 secretion was observed in the PPL-C group (**Figure 8G**). Importantly, the decrease in IL-10 was associated with counteracting Treg differentiation. We speculate that the reduction of PD-L1+ Tregs in the PPL-C group resulted in fewer IL-10-producing Tregs. These findings are similar to previous studies that suggest compensatory IL-10 release as one of the adaptive resistance mechanisms undermining the effectiveness of anti-PD-L1 monotherapies (22). IFN-γ, TNF and IL-2 were found to be considerably increased by PPL-C when we assessed the cytokine concentration in cell culture supernatants, which further demonstrated that lymphocytes in immunotherapy-treated mice have been reactivated by tumor cells (**Figures 8A–F**). These results indicated the cytotoxicity and reactivation of PPL-C peptide-stimulated PBMCs in immunotherapy-treated mice. Collectively, our findings suggested the possibility for PPL-C peptides to be developed as low molecular weight therapeutic options for cancer immunotherapy.

### Prediction of potential PPL-C to PD-L1 binding sites

The 3D spatial structure prediction of PPL-C using LOMETS revealed a composition consisting of a random coil and beta-sheet, as depicted in **Supplementary Figure S12A**. Additionally, the ZDOCK analysis predicted the docking model of PPL-C to PD-L1 (PDB ID: 3BIS), indicating that the docking site of PPL-C might be located close to the PD-1 and PD-L1-interaction pocket, as shown in **Supplementary Figures S12B, C**, and the binding position of PD-1 (light sea green) to PD-L1 (dark khaki, represented by a space-fill model) is also illustrated. These findings, in conjunction with the ligand inhibition assay results, suggest that the binding site of PPL-C is likely in close proximity to the PD-1 and PD-L1-binding pocket.

## Discussion

Immunotherapy with anti-PD-1 (programmed cell death protein 1) antibodies is a significant component of immune checkpoint inhibitor therapy (23). A key immunosuppressive molecule PD-1 is a membrane protein with 288 amino acid residues that belongs to the immunoglobulin superfamily (23). Organ transplant recipients survival and the treatment of autoimmune, infectious, and malignant disorders all significantly impact immunomodulation that targets PD-1. A PD-L1 ligand can be a target and the corresponding antibody can serve the same purpose. When PD-1 and PD-L1 integrate, T cells experience programmed death, which allows tumor cells to evade the immune system. The PD-1 antibody can block PD-1 and PD-L1 from binding, which activates immune cells and destroys tumors (24). There are 6 PD-1 inhibitors available or FDA-approved, which have significantly altered tumor treatment. Anti-PD-1 treatment offers outstanding therapeutic results, although it struggles with poor clinical response rates.

In the present study, we screened PD-L1-targeting peptides using phage display technology (5). We successfully identified a group of candidate peptides (PL-8, PL-20, and PPL-C), among which PPL-C showed high-affinity binding to cell-surface-expressed PD-L1. We developed a PD-1 antagonist peptide PPL-C employing phage display evolution potential. PPL-C can block PD-1/PD-L1 binding and regenerate the T cells defensive potential *in vitro*. Competitive ELISA confirmed that PPL-C block the interaction between PD-L1 and PD-1. PPL-C specifically binds to murine PD-L1 and also binds to human PD-L1. According to ZDOCK software predictions, the PPL-C binding site to PD-L1 is close to the PD-1 and PD-L1 interaction sites, suggesting that PPL-C may operate as a blocker molecule by hindering PD-1 ability to connect to PD-L1 (5). Earlier experiments have demonstrated the significant role of PD-L1 as a tumor or antigen-presenting cell-associated negative regulator of CD4+ T cell responses (25, 26). The inhibiting impact of PPL-C on PD-1/PD-L1 interactions was explained by computer docking, which indicated that the PPL-C interaction site on PD-L1 overlapped with the PD-1/PD-L1 interface. In this study, phage display was used to identify many peptide sequences that target PD-L1, and we noted the conserved region at the N-terminus. This was further confirmed by the docking result that PPL-C binds with PD-L1 to inhibit its activity.

The most effective immune cell type for eliminating tumor cells is CD8+ T lymphocytes (CTLs). Due to immune-related tolerance and immunosuppression inside TME, CTLs experience malfunction and exhaustion throughout cancer development (27). Under normal conditions, Tregs express 4-5% of the entire CD4+T cell population (28). As Tregs are the dominant T cells that react to malignancies and outpace CTLs during early cancer progression, an increase in their number is prevalent in cancer patients (29, 30). Cancer treatment strategies targeting Tregs are currently a research hotspot. The PPL-C discovered in this manuscript effectively inhibits Tregs differentiation, reactivates T cells, and achieves effective tumor killing. By FCM, we evaluated that PPL-C could enhance the concentration of CD8+ T cells and decline the concentration of Treg cells in TILs, thus, elevating the concentration of CD8+ T cells to Treg cells. This will further promote the development of tumor immunotherapy that targets Tregs clearance and controls Treg cell activity and infiltration. The immunological balance in the TME is represented by the ratio of CD8+ T cells to Tregs, which has been utilized as a prognostic indicator. Tregs are also associated with poor prognosis in solid cancers, suggesting their importance in cancer treatment (31). Therefore, regulating Tregs or their signaling pathways to reverse the immunosuppressive microenvironment will have a positive effect on tumor treatment. Our immunohistochemistry study confirmed these outcomes. Additionally, IHC also noted an increase in CD4+ T cells in malignancies. By facilitating CD8+ T cell priming or migration to the tumor site, CD4+ T cells contribute to tumor rejection (32). By using immunofluorescence, PPL-C therapy boosted IFN-γ release by tumor-infiltrating T cells, demonstrating that PPL-C restored T lymphocyte activity in TME. All of these findings support PPL-C’s ability to rebalance immunosuppressive TME and enhance the antitumor response.

As we all know, the activation of T lymphocytes is a crucial step in the immune response. The induction of T cell activation and proliferation requires two signals: the first signal is the specific antigen stimulation signal generated by the T cell receptor (TCR) on the surface of T lymphocytes binding to the MHC-antigen complex. The second signal is a non-specific costimulatory signal, mainly generated by the interaction between multiple pairs of costimulatory molecules on the surface of APCs and the corresponding receptors on T cells (such as: CD28, CTLA-4 and CD80, CD86, 4-1BB and 4-1BBL, CD40 and CD40L, PD-1 and PD-L1, etc.), among which CD28 is the most important costimulatory molecule. After being stimulated by this signal, T cells become fully activated, secreting a large number of cytokines (such as IL-2) and expressing cytokine receptors, ultimately exerting the immune killing function. The T-cell activation assay stimulates T cells by combining CD3 and CD28 antibodies *in vitro*, simulating the dual signal effect of T cell activation *in vivo*. It is currently the most extensive method for studying T cell activation and expansion, and is also an important method for evaluating the effects of immunotherapy drugs (10). Here, we used IFN-γ expression to represent T-cell activation. Studies have shown that PD-L1 inhibitor treatments promote IFN-γ production, which in turn eliminates cancer cells. Tumor-infiltrating CD8+ T lymphocytes are one of the primary sources of IFN-γ in tumors (11). PPL-C can improve CD4+ and CD8+ T cells performance *in vitro* PBMC experiments when stimulated by anti-CD3/anti-CD28. However, it will be more natural and realistic to identify the impact of PPL-C on T cell activity in the tumor-bearing mouse model without anti-CD3/anti-CD28. Upon binding to the PD-L1 protein, PPL-C disrupts the interaction between PD-1/PD-L1, resulting in an antigen-specific CD8+ T cell response, reactivating CD8+ T cells, and ultimately killing the tumor cells. We identified that PPL-C could overcome PD-1 inhibitory effects and increase IFN-γ production during naïve CD4+ T cell activation.

Experiments conducted on tumor-bearing mouse models demonstrated that the peptides inhibited tumor growth and prolonged animal survival. The size of tumors and tumor masses did not grow as quickly in the PD-L1 mAb and PPL-C groups as they did in the control peptide and untreated groups. This shows that T cells were activated again and affected cancer cells cytotoxically. PD-1 was particularly increased in drained T cells (12). The drained CD8 T cells responded favorably to the blockade of the PD-1/PD-L1 inhibitory pathway, regaining the capacity to multiply release cytokines and improve T-cell responses (13). We observed that PPL-C therapy might increase the number of CD8+ T cells at the tumor site in CT26 tumor-bearing mice, which may be due to CD8+ T cell proliferation. However, the number of CD4+ T cells did not change much. Previous studies revealed that PD-1+ CD8+ T cells after chronic LCMV (lymphocytic choriomeningitis virus) infection resembled stem cells, and the proliferative CD8+ T cells following PD-1 inhibition were largely generated from this CD8+ T-cell fraction (14). Similar results were observed in our study, which showed that PPL-C activated the proliferation of PD-1+ CD8+ but not PD-1+ CD4+ T cells in tumors by blocking PD-1/PD-L1. This supports the idea that CD8+ T cells are more crucial to the antitumor response than CD4+ T cells.

Additionally, it should be noted that the NK T cell frequency in the immunotherapy groups, such as the *in vivo* MAb anti-mouse PD-L1 group, was much lower than that of the control peptide or PBS group. This reveals that PPL-C is an antagonistic mAb without antibody-dependent cell-mediated cytotoxicity (ADCC) and complement-dependent cytotoxicity (CDC), which has the potential to be a therapeutic antibody drug for cancer. Therefore, these mAbs should be designed or engineered to eliminate ADCC, which has been identified as a crucial mechanism in several extremely successful MAb-mediated cancer treatments. A completely human anti-PD-L1 mAb could prevent the interaction between PD-L1 and PD-1 in addition to promoting the ADCC lysis of tumor cells. However, combining our peptides with medications that target checkpoints or other therapies, such as conventional chemotherapy or radiation may be a preferable option. Our study showed that PPL-C peptide at higher concentrations inhibited tumor development like PD-L1 mAb. Cancers, allergic illnesses, infectious diseases, autoimmune diseases, fibrosis, and asthma have all shown benefits from peptide-based treatment. A growing number of clinical studies have been carried out on many additional cancer types, including melanoma, glioblastoma, breast cancer, and gastric cancer since the FDA authorized sipuleucel-T as the first peptide vaccine for prostate cancer. However, challenges still exist in the development and wide application of peptide drugs. Peptides have a relatively short circulating plasma half-life and may undergo enzymatic degradation in plasma, as well as fast renal clearance when injected intravenously. To overcome these limitations, researchers have designed nanocarriers for peptide delivery and conjugated structure-inducing probes (SIP)-tail, lactam bridges, and stapling or clipping of peptide sequences or cyclization to avoid enzymatic digestion and prolong circulation time. Moreover, emerging technologies, such as dual or even triple agonism multifunctional peptides, offer innovative opportunities for peptide drug development. We believe that peptides as drugs will continue to be widely used. By disabling the PD-1/PD-L1 pathway, PD-L1 binding peptides can significantly reverse immunological suppression and enable T cell effector activity against different malignancies.

## Conclusion

By applying mirror-image phage display technology, we discovered a novel peptide, PPL-C, which binds exclusively to PD-L1. Specifically, PPL-C demonstrated remarkable inhibitory effects on tumor growth in a mouse model in which CT26 cells were transplanted. In comparison with mice treated with a control peptide, the growth rate of tumor masses treated with PPL-C and PD-L1 antibodies was significantly reduced by 78% and 77%, respectively. The PPL-C gene has been shown to enhance the cytotoxicity of lymphocytes in CT26-bearing mice, which results in a more refined suppression of tumor growth. Furthermore, it promotes the proliferation of lymphocytes, which contributes to the enhancement of the immune response against the tumor. Considering its potential as a cancer immunotherapy drug candidate, PPL-C warrants further research and development.

## Future recommendations

To facilitate the early clinical application of PPL-C, additional research is needed in the following areas:

- Optimization of phage screening technology, design of negative control phages, and analysis of their affinity with PD-L1 to further clarify the specificity of PPL-C’s interaction with PD-L1.
- Utilization of the CRISPR-Cas9 system to knock out the PD-L1 or PD-L2 gene for further verification of PPL-C’s affinity specificity.
- Performance of crystal structure diffraction analysis of mutations in the protein binding sites of PPL-C and PD-L1 to clarify the specific binding site.
- Conducting pharmacokinetics and tissue enrichment experiments, including blood concentration detection, tumor tissue enrichment *in vivo* imaging, serum stability testing, and enzyme activity stability testing to verify *in vivo* stability.
- Optimization of experimental concentration and treatment time in protein and antibody competition experiments to clarify the concentration-dependent relation of the affinity between PPL-C and PD-L1.
- Investigation of the synergistic effect of PPL-C on the production of IgG antibodies induced by heterologous antigens *in vivo* and exploration of its immune synergistic effect.
- Exploration of the therapeutic effects of PPL-C on CT26 colon cancer in different stages and verification using other tumor models or combination with other antibody drugs or inhibitors. Additionally, studying the tumor inhibitory effect and mechanism of PPL-C using humanized mice.
- Verification of the biological activity of PPL-C in blocking PD-1/PD-L1 interaction using human PBMC or human cell lines expressing PD-1 or PD-L1.
- Design of a multi-epitope vaccine containing PPL-C to explore its adjuvant and immune synergistic effect. Conducting pharmacodynamic research on PPL-C by enhancing the immunogenicity of CT26 tumor cell antigen.
- Study of specific changes in lymphocyte subpopulations and cytokine expression in the immune organs and tumor microenvironment of mice after PPL-C treatment to further understand its mechanism of action and potential risks.

## Data availability statement

The original contributions presented in the study are included in the article/**Supplementary Material**. Further inquiries can be directed to the corresponding authors.

## Ethics statement

The animal study was approved by Laboratory Animal Ethics Committee, School of Life Sciences, Fudan University. The study was conducted in accordance with the local legislation and institutional requirements.

## Author contributions

YL: Investigation, Methodology, Validation, Writing – original draft, Writing – review & editing. HL: Investigation, Methodology, Validation, Writing – original draft. XL: Investigation, Methodology, Writing – original draft. SL: Writing – review & editing. AH: Writing – review & editing. BL: Investigation, Methodology, Writing – original draft. JH: Investigation, Methodology, Writing – original draft. JW: Investigation, Methodology, Writing – original draft. HY: Investigation, Methodology, Writing – original draft. BH: Investigation, Methodology, Writing – original draft. LZ: Investigation, Methodology, Writing – original draft. JX: Conceptualization, Project administration, Supervision, Writing – original draft. NZ: Conceptualization, Funding acquisition, Project administration, Supervision, Writing – original draft.
