# Supplementary material for: PD-L1 targeted peptide demonstrates potent antitumor and immunomodulatory activity in cancer immunotherapy
Source: Front Immunol. 2024 Apr 30;15:1367040. doi: 10.3389/fimmu.2024.1367040 (PMC11091243; doi:10.3389/fimmu.2024.1367040)
Supplement: Supplementary file 1 [file DataSheet_1.docx]

**Supplemental Material**


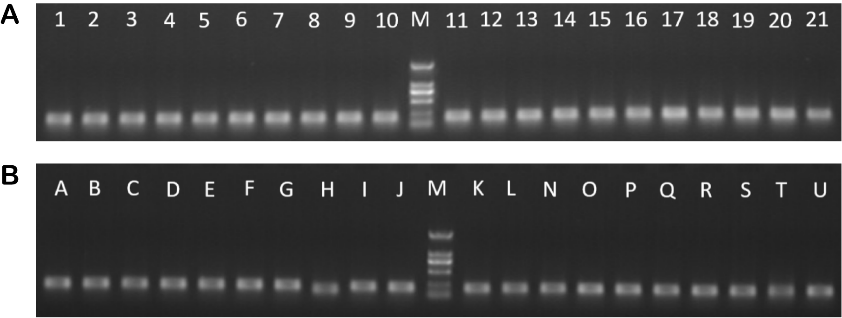


Figure. S1. Agarose gel electrophoresis of PCR reaction solution from Phage cloning. To investigate whether the selected phage has wild-type contamination, we picked 21 clones, taking 1μl as a template for PCR, and ran 1% agarose gel electrophoresis. M stands for Marker. The numbers indicate the PCR reaction solution.


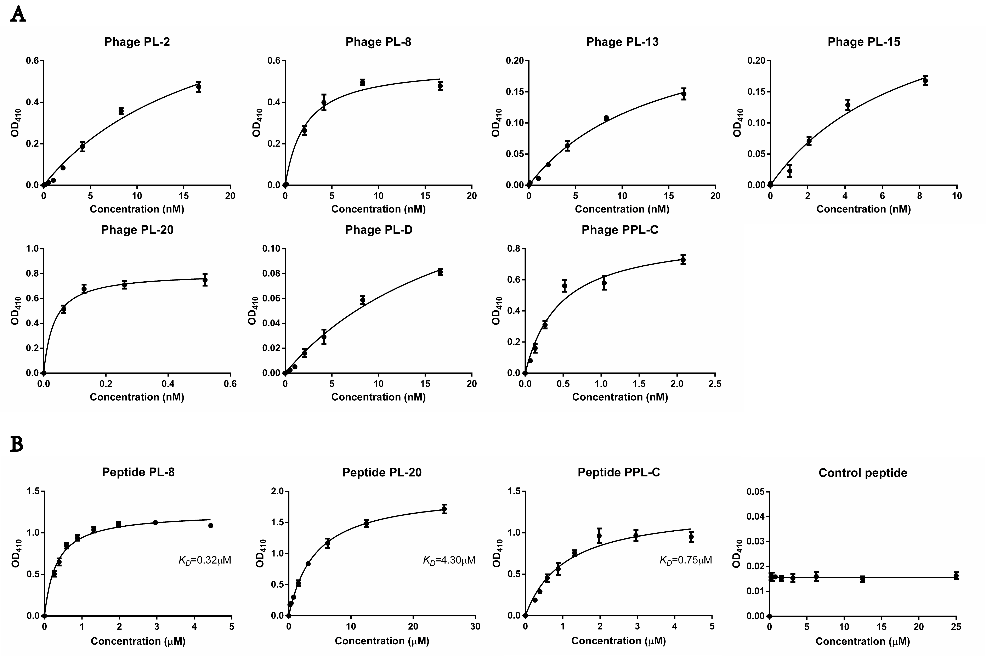


Figure. S2 A. Binding properties of peptides displayed on the bacteria surface. The binding ability of the clone phage PL-2, phage PL-8, phage PL-13, phage PL-15, phage PL-20, phage PL-D, and phage PPL-C to PD-L1 at different phage concentrations by ELISA assay (nM, n = 3). **B.** Binding specificity of peptides PL-8, PL-20, PPL-C, and Control peptide to PD-L1 at different peptide concentrations by ELISA assay ($\mu$M, n = 3). Data were presented as mean ± SEM.


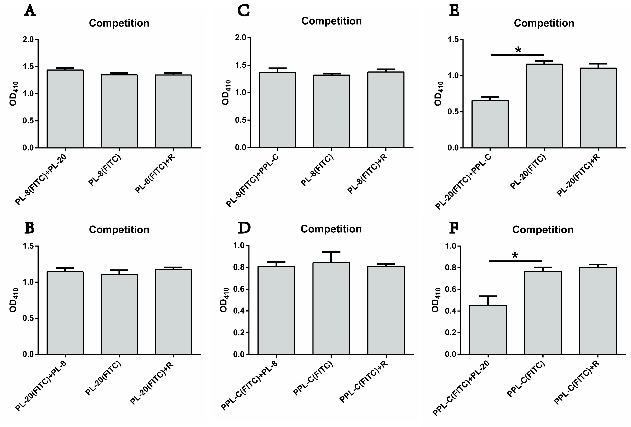


Figure. S3. ELISA assay for the competition of three candidate peptides for PD-L1 affinity sites. A, B. PL-8, and PL-20 competition. C, D. PL-8, and PPL-C competition. E, F. PL-20, and PPL-C competition. Data, mean $\boldsymbol{\pm}$ SEM; *, P **<** 0.05.


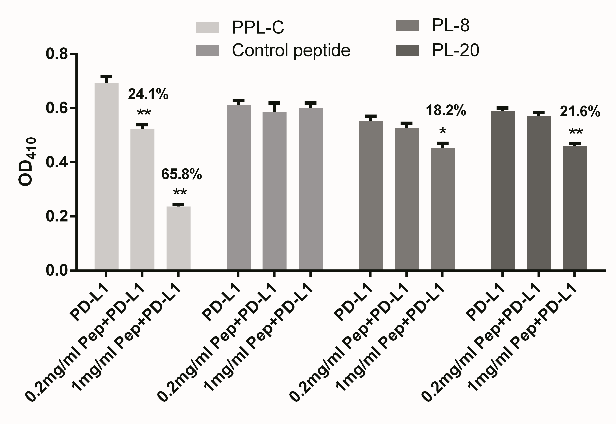


Figure. S4. The competition of PD-L1 binding to PD-1 by PPL-C, PL-8, PL-20, and control peptides was detected by ELISA (n = 3). The percentage on the histogram shows the degree of down-regulation of PD-1 and PD-L1 binding. Data, mean $\boldsymbol{\pm}$ SEM; *, P **<** 0.05; **, P **<** 0.01.


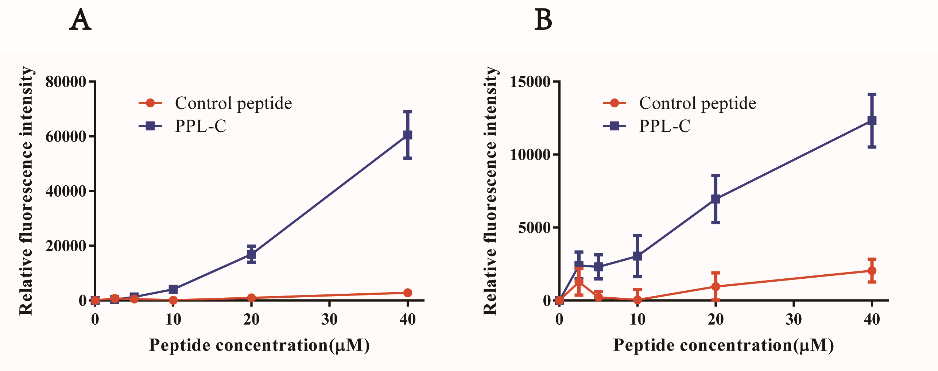


Figure. S5 A. The binding properties of PPL-C and control peptide to mPD-L1 were determined by the ELISA (n = 3). B. The binding properties of PPL-C and Control peptide to hPD-L1 were determined by the ELISA (n = 3). Data, mean $\boldsymbol{\pm}$ SEM; *, P **<** 0.05; **, P **<** 0.01.


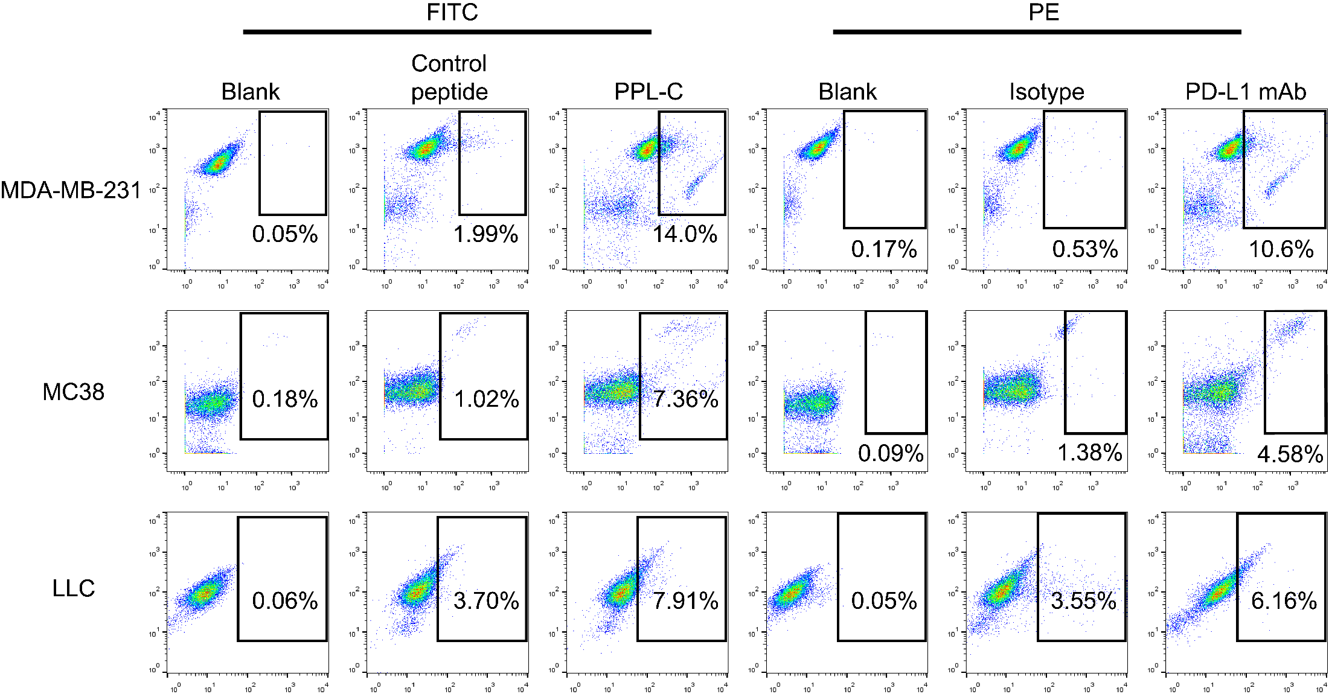


Figure. S6. The flow cytometric determination of PPL-C specifically targeting PD-L1-expressing cell lines and the competition for PD-L1 monoclonal antibodies. The representative plots show the percentages of the detection of PD-L1 positive rates by peptides and antibodies. The binding properties of PPL-C to MDA-MB-23, MC38, and LLC cell lines were determined by flow cytometry and statistical analysis of the results of three repeated flow experiments.


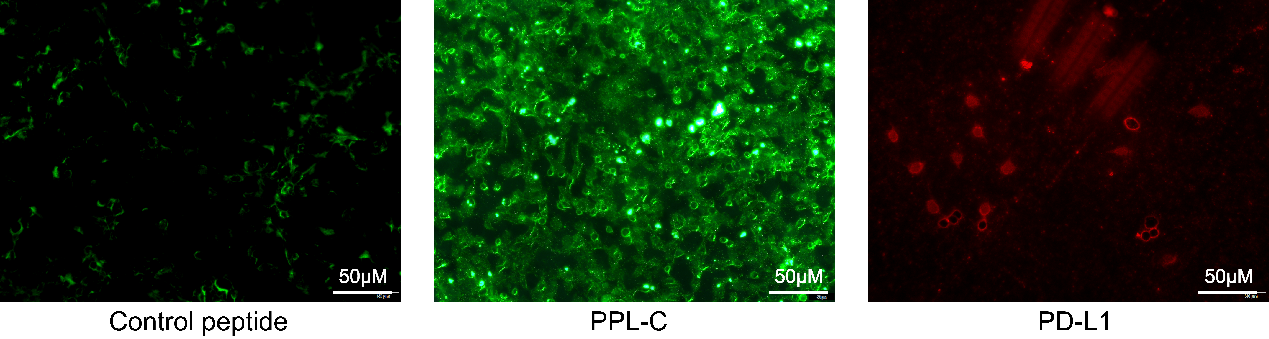


Figure. S7. The fluorescence microscopic image of PPL-C to LLC. Cells fixed with paraformaldehyde were incubated with FITC-conjugated PPL-C and PE-conjugated PD-L1 mAb, and then observed by electron microscopy.


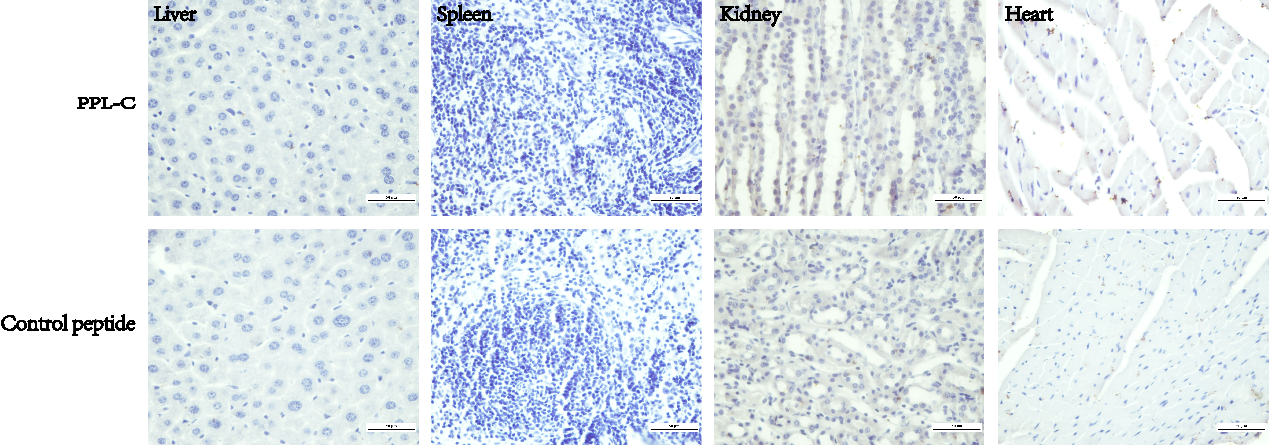


Figure. S8. Non-specificity of PPL-C immunostaining for normal tissues, representative images of four negative immunohistochemical staining results indicate non-specific binding to normal tissue.


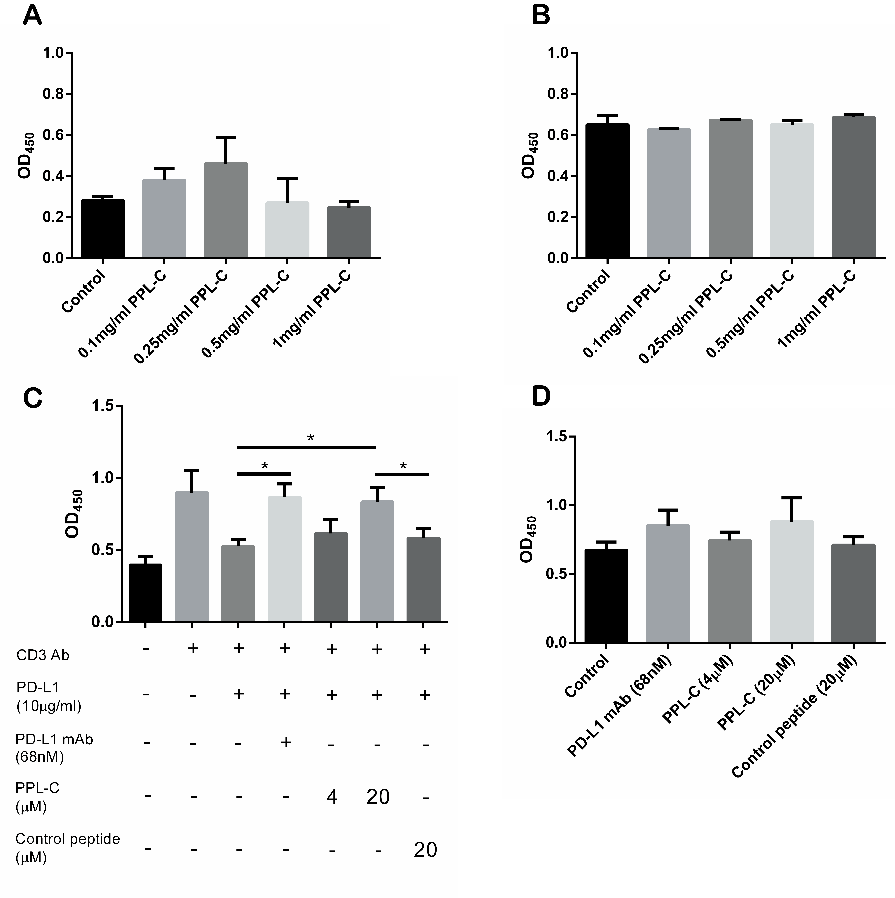


Figure. S9. OD_450_ of CT26 cells after incubation with different concentrations of PPL-C for 8 (**A**) (n = 3) and 24 (n = 3) hours (**B**) by CCK-8 cell viability assay (OD=optical density). **C.** PPL-C could effectively restore T cell proliferation which was inhibited by PD-L1 evaluated by T cell activation assay (n = 3). **D.** The proliferation of T cells in MLR assay (n = 3). Data were presented as mean ± SEM, *, P<0.05.


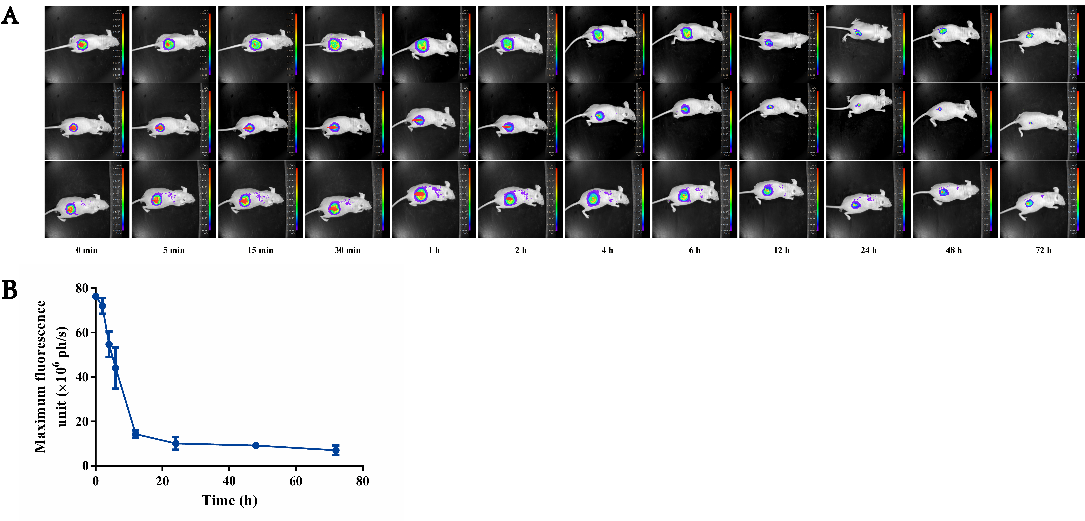


Figure. S10. A. In vivo fluorescence images of mice taken at different time points post s.c. injection of FITC labeled PPL-C. **B.** The statistical result of the relative maximum fluorescence unit (n = 3). Data were presented as mean ± SEM.


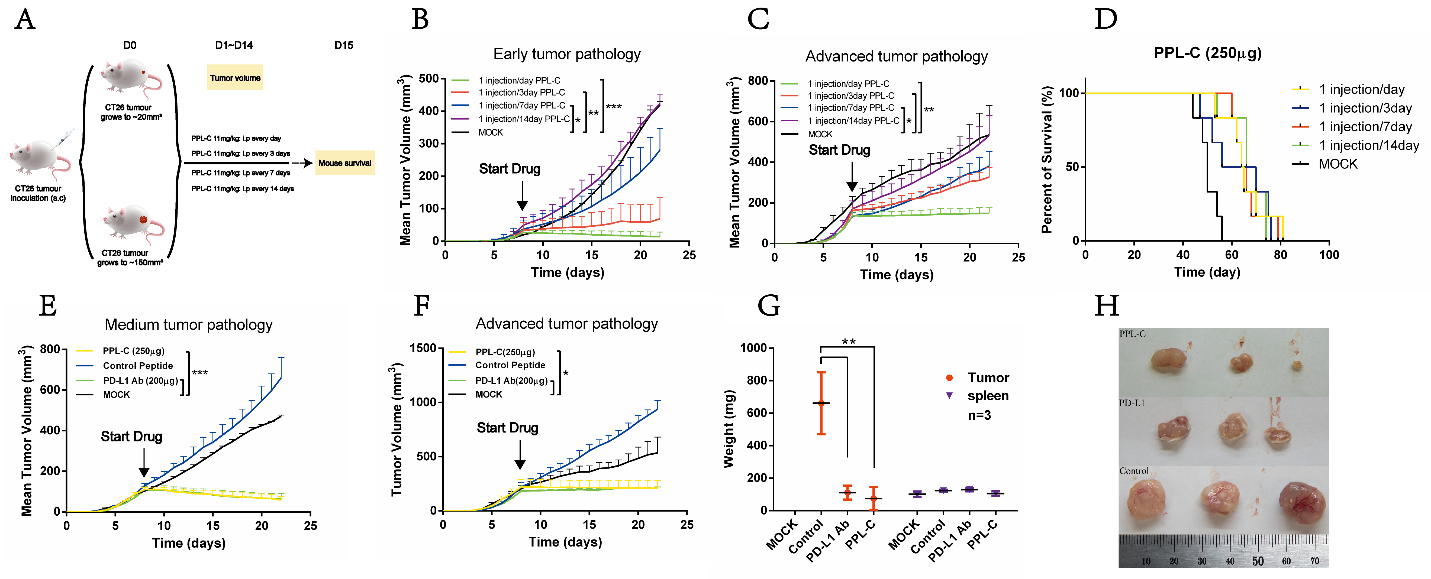


Figure. S11. Exploration of PPL-C Peptide Dosage in CT26 Tumor-bearing Mice**. A.** Treatment schedule for the immunotherapy. **B.** Tumor growth inhibition in CT26-tumor-bearing Balb/c mice injected with PPL-C subcutaneously around the tumor at early tumor pathology (n = 6) or advanced tumor pathology (C) (n = 6). Significant differences in the tumor volume between the PPL-C group and the negative control were determined by student’s t-test, *, P **<**0.05; **, P**<** 0.01; ***, P**<** 0.001. D. Survival prolonging curves for CT26-tumor-bearing Balb/c mice treated with different doses of PPL-C. E, F. Growth inhibition of the medium tumor pathology or Advanced tumor pathology via PPL-C (n = 3). G, H. CT26 tumor tissue and spleen tissue anatomically implanted in Balb/c mice were weighed after different treatments, and representative images of tumors at the end of treatment in CT26 tumor-bearing mice. Data, mean $\boldsymbol{\pm}$ SEM; *, P **<** 0.05; **, P **<** 0.01.


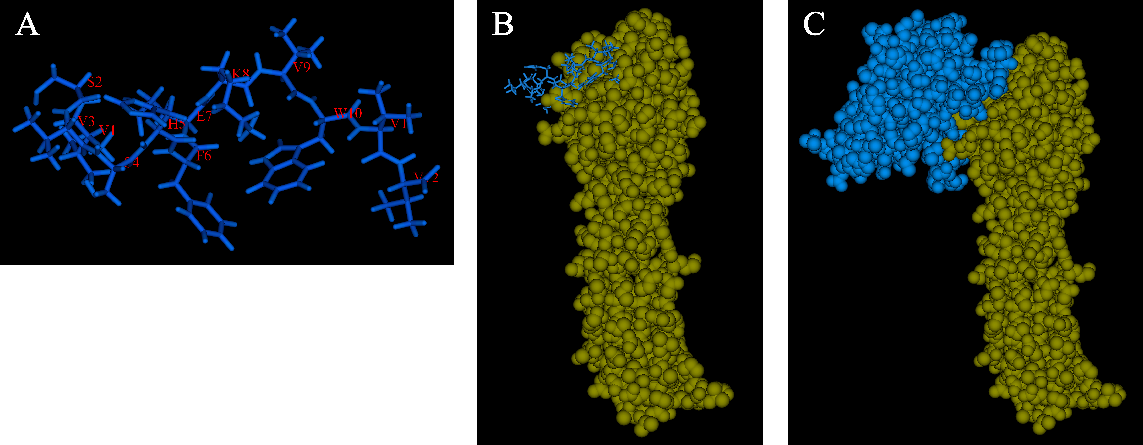


Figure. S12. A. Analysis of three-dimensional space structure diagram of mimic peptide PPL-C with the van der Waals, letters and numbers indicate amino acids and positions. B. The possible docking model of PPL-C (blue) peptide to PD-L1 (PDB ID of PD-L1: 3BIS) complexes. C. The crystal structures of PD-1 (light sea blue) and PD-L1 (dark khaki) complex (PDB ID of the complex: 3BIK).

Table S1. Phage display library screening sequence

| Name | Sequence |
| --- | --- |
| PL-2 | HIGTIPEGRAFH |
| PL-8 | HTSWILYGETGW |
| PL-13 | GLFGNEARTAST |
| PL-15 | HISYEIGLRPGD |
| PL-20 | HESWLPAYVLGS |
| PPL-C | VSVSHFQKVWVV |
| PL-D | TSQTNAKVWQIY |

Seven fragments of the peptide that may be affinity to the PD-L1 protein are screened by the phage display library.

Table S2. Candidate peptides with good affinity for PD-L1 protein

| Name | Sequence (N$\to$C) | Purity |
| --- | --- | --- |
| PPL-C | SVSVSHFQKVWVVGGGSK-NH_2_ | $>$98% |
| PPL-C (FITC) | SVSVSHFQKVWVVGGGSK-FITC-NH_2_ | $>$98% |
| PL-20 | SHESWLPAYVLGSGGGSK-NH_2_ | $>$98% |
| PL-20 (FITC) | SHESWLPAYVLGSGGGSK-FITC-NH_2_ | $>$98% |
| PL-8 | SHTSWILYGETGWGGGSK-NH_2_ | $>$98% |
| PL-8 (FITC) | SHTSWILYGETGWGGGSK-FITC-NH_2_ | $>$98% |
| Control peptide | SMQYADHPVNTGGGGSK-NH_2_ | $>$98% |
| Control peptide (FITC) | SMQYADHPVNTGGGGSK-FITC-NH_2_ | $>$98% |

Table S3. Peptide PPL-C as an inhibitory candidate for PD-L1 to treat cancers

| Name | Sequence (N$\to$C) | Purity |
| --- | --- | --- |
| PPL-C | SVSVSHFQKVWVVGGGSK-NH_2_ | $>$98% |
| PPL-C(PEG8) | SVSVSHFQKVWVVGGGSK(PEG8)-NH_2_ | $>$98% |
| PPL-C (FITC) | SVSVSHFQKVWVVGGGSK-FITC-NH_2_ | $>$98% |
| Control peptide | SMQYADHPVNTGGGGSK-NH_2_ | $>$98% |
| Control peptide(PEG8) | SMQYADHPVNTGGGGSK(PEG8)-NH_2_ | $>$98% |
| Control peptide (FITC) | SMQYADHPVNTGGGGSK-FITC-NH_2_ | $>$98% |
